# First Precambrian palaeomagnetic data from the Mawson Craton (East Antarctica) and tectonic implications

**DOI:** 10.1038/s41598-018-34748-2

**Published:** 2018-11-06

**Authors:** Yebo Liu, Zheng-Xiang Li, Sergei A. Pisarevsky, Uwe Kirscher, Ross N. Mitchell, J. Camilla Stark, Chris Clark, Martin Hand

**Affiliations:** 1 Earth Dynamics Research Group, ARC Centre of Excellence for Core to Crust Fluid Systems (CCFS) and The Institute for Geoscience Research (TIGeR), School of Earth and Planetary Sciences, Curtin University, GPO Box U1987, Bentley, WA 6845 Australia; 2 School of Earth and Planetary Sciences, Curtin University, GPO Box U1987, Bentley, WA 6845 Australia; 3 Department of Earth Science, School of Physical Sciences, University of Adelaide, Adelaide, South Australia 5005 Australia

## Abstract

A pilot palaeomagnetic study was conducted on the recently dated with *in situ* SHRIMP U-Pb method at 1134 ± 9 Ma (U-Pb, zircon and baddeleyite) Bunger Hills dykes of the Mawson Craton (East Antarctica). Of the six dykes sampled, three revealed meaningful results providing the first well-dated Mesoproterozoic palaeopole at 40.5°S, 150.1°E (A_95_ = 20°) for the Mawson Craton. Discordance between this new pole and two roughly coeval poles from Dronning Maud Land and Coats Land (East Antarctica) demonstrates that these two terranes were not rigidly connected to the Mawson Craton ca. 1134 Ma. Comparison between the new pole and that of the broadly coeval Lakeview dolerite from the North Australian Craton supports the putative ~40° late Neoproterozoic relative rotation between the North Australian Craton and the combined South and West Australian cratons. A mean ca. 1134 Ma pole for the Proto-Australia Craton is calculated by combining our new pole and that of the Lakeview dolerite after restoring the 40° intracontinental rotation. A comparison of this mean pole with the roughly coeval Abitibi dykes pole from Laurentia confirms that the SWEAT reconstruction of Australia and Laurentia was not viable for ca. 1134 Ma.

## Introduction

**Figure 1:**
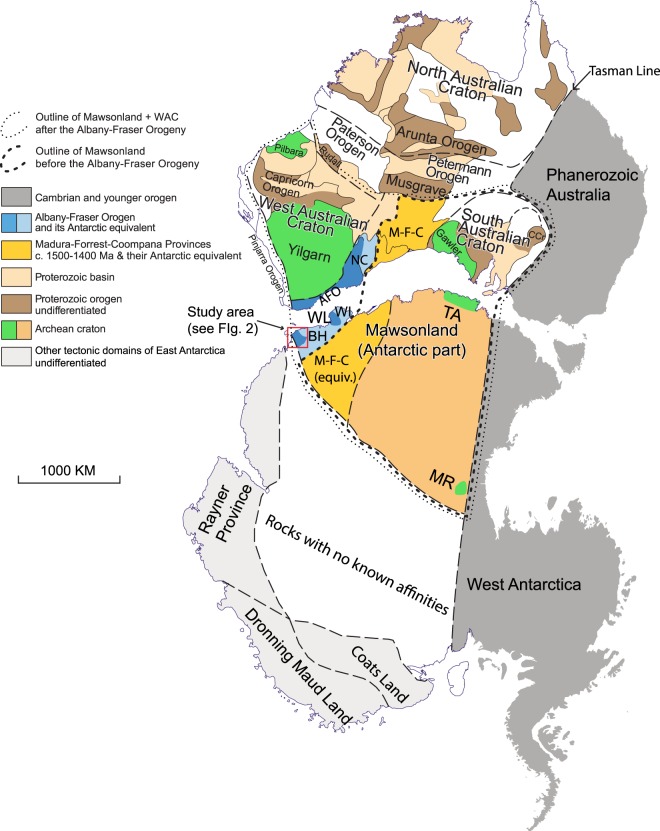
Tectonic map of Australia and Antarctica in a Gondwana configuration (modified after ref.^75^ with data incorporated from refs^16,76^). Antarctica is rotated to Australia coordinates using a Euler pole^7^ at 1.3°N, 37.7°E, rotation = 30.3°. Abbreviations: AFO, Albany-Fraser Orogen; BH, Bunger Hills; CCr, Curnamona Craton; M-F-C, Madura-Forrest-Coompana Provinces; MR, Miller Range; NC, Nornalup Complex; TA, Terre Adélie craton; WI, Windmill Islands; WL, Wilkes Land.

East Antarctica has been a key piece in Precambrian palaeogeographic reconstructions (e.g., refs^1–4^). Nevertheless, available constraints for Precambrian palaeogeography for East Antarctica are quite sparse for several reasons: (i) logistical inaccessibility, (ii) limited outcrops due to the thick ice cover, and (iii) difficulties in conducting fieldwork in the severe weather. There are only two Precambrian palaeomagnetic poles available from East Antarctica: the ca. 1130 Ma pole from the Borgmassivet intrusions in Dronning Maud Land^5^ and the ca. 1100 Ma “CL” pole from Coats Land^6^ (BM and CL hereafter). However, it is likely that neither Dronning Maud Land nor Coats Land terranes joined the Mawson Craton until the final assembly of Gondwana ca. 520 Ma^1,3,7–10^. Therefore, the BM and CL poles cannot be used to constrain the location of the Mawson Craton in pre-530 Ma palaeogeographic reconstructions. As a result of both the lack of palaeomagnetic data from the Mawson Craton (East Antarctica) and the long-lived connection between Mawson and Gawler (South Australia) cratons (comprising the so-called Mawsonland; Fig. 1), the placement of East Antarctica in Precambrian palaeogeographic reconstructions has relied indirectly on the dataset of Australia in an assumed Gondwanan configuration (e.g., refs^4,11,12^).

The Bunger Hills area of the Wilkes Land district of East Antarctica is commonly considered to be a fragment of the Archaean Yilgarn Craton^13–15^ (Fig. 1). Bunger Hills became a part of the Mawsonland during the ca. 1.3 Ga Albany-Fraser Orogeny^15–18^. Following the Ectasian orogenesis, Bunger Hills was intruded by abundant mafic dykes that can be divided into two groups: an older, deformed and metamorphosed dykes, and a younger, non-deformed and non-metamorphosed dykes. In this study we dealt with the second group only. These non-deformed dykes were classified into five compositionally distinctive sub-groups ranging from olivine tholeiites and slightly alkaline dolerites to picrites–ankaramites^19^. Those five sub-groups were proposed to have reflected lateral and vertical heterogeneity in their source regions and indicated the involvement of at least six different source regions of mantle partial melt^19^. One sub-group probably originated from an enriched lithospheric mantle source with an OIB-like component, whereas other dyke groups likely had at least two source components ranging from slightly depleted to moderately enriched in composition. Geochemical analysis of the largest ~50-m-wide dyke at Bunger Hills (sample BHD1) supports this conclusion^20^.

Whole-rock Rb–Sr and Sm–Nd mineral isochron dating suggests emplacement of the tholeiites and dolerites at ca. 1140 Ma and the alkali dykes at ca. 502 Ma^19,21,22^. The 6 dykes sampled for this study are all roughly NW-trending dolerites or gabbros. Among them, BHD1, the largest NW-trending dyke at Bunger Hills, has recently been dated with *in situ* SHRIMP at 1134 ± 9 Ma (zircon) and 1131 ± 16 Ma (baddeleyite), suggesting that similarly oriented dykes with ca. 1140 Ma Rb-Sr and Sm-Nd dates may be coeval^20^. In this paper, we present the results of a palaeomagnetic study of these ca. 1134 Ma Bunger Hills mafic dykes, representing the first Precambrian palaeomagnetic pole from the Mawson Craton of East Antarctica, and discuss its tectonic implications.

## Methods

**Figure 2:**
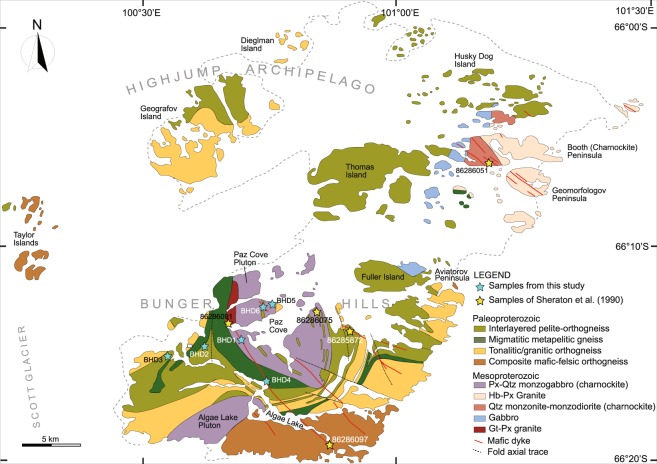
Simplified geological Map of Bunger Hills showing the sample locations (modified after ref.^20^).

A total of 36 block samples from 6 sites (6 dykes, including the recently dated BHD1 dyke) were collected for palaeomagnetic analysis (Fig. 2). All samples were oriented with both a magnetic compass and a sun compass, except those from dyke BHD3 where only magnetic compass was used due to weather conditions. At least two cylindrical specimens were drilled from each block. At least one specimen per block was subjected to progressive thermal demagnetisation in 15 to 20 steps from 100 °C to 600 °C using a Magnetic Measurements Ltd thermal demagnetiser. After each heating step, the magnetisation was measured using an AGICO JR-6A spinner magnetometer. An initial set of samples was also subjected to alternating field (AF) demagnetisation and measurement using the 2 G RAPID system with maximum AF fields of 110 mT. Both magnetometers are hosted inside the magnetically shielded room.

Magnetisation vectors were defined using principal component analysis^23^. All vectors were calculated using at least four successive steps with maximum angular deviations <10°. In cases where demagnetisation failed to reveal stable endpoints, remagnetisation great circles were used^24^. Site-mean directions were calculated in these cases using the method described in ref.^25^. Mean dyke directions were calculated using Fisher statistics^26^. All calculations were carried out using PuffinPlot^27^ and the PmagPy package^28^. GPlates software^29^ was used for palaeogeographic reconstruction.

To identify the magnetic carrier(s) for the various isolated components, samples with representative demagnetisation behaviour were each given a three-component isothermal remanent magnetisation (IRM) along three orthogonal axes using magnetic fields of 2.4 T, 0.4 T and 0.12 T, respectively^30^, using a Magnetic Measurement MMPM10 pulse magnetiser. The IRMs were then subjected to progressive thermal demagnetisation. Susceptibility versus temperature experiments were conducted using an AGICO MFK-1 Kappabridge (equipped with a CS4 furnace). Hysteresis loops and isothermal remanent magnetization curves were obtained with a Variable Field Translation Balance (VFTB^31^). All the measurements were carried out in the palaeomagnetism laboratory at Curtin University.

## Results

### Rock magnetism

**Figure 3:**
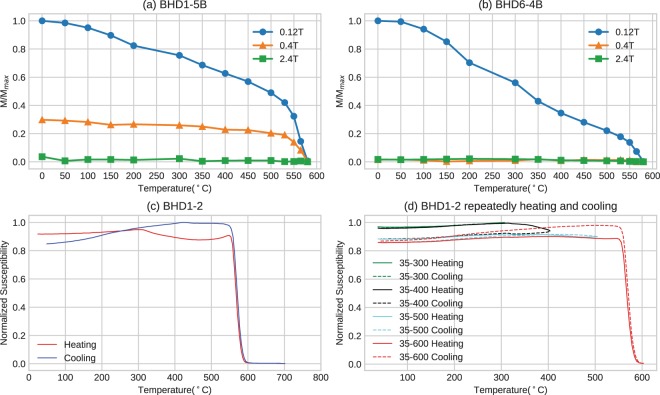
Results of thermomagnetic experiments on representative dyke samples. (**a**,**b**) Thermal demagnetisation of orthogonal three-axis IRMs; (**c**,**d**) temperature versus susceptibility curves.

The results of the Lowrie^30^ test show that the low-coercivity fraction (0–0.12 T) with Curie temperatures of ~580 °C is dominant in all tested specimens and is probably carried by multi-domain low-titanium titanomagnetite or magnetite (Fig. 3). The medium-coercivity fraction (0.4 T) with Curie temperatures of ~580 °C is also significant in most tested specimens, suggesting the additional presence of palaeomagnetically highly stable single-domain (SD) or pseudo-single-domain (PSD) (titano)magnetite (Fig. 3a). In one case (specimen BHD6–4B), only multi-domain magnetite is present (Fig. 3b).

Susceptibility versus temperature curves (Fig. 3c,d and Supplementary Fig. 2) show consistent sharp declines in susceptibility between 560 °C and 590 °C, indicating that the main magnetic mineral phase is Ti-poor titanomagnetite/magnetite. Hopkinson peaks^32,33^ are observable in some samples (Fig. 3c and Supplementary Fig. 2b,c) suggesting the presence of single domain (titano)magnetite. In all measured samples, a decrease in intensity during heating starting from 320 °C disappears during cooling, which implies the occurrence of a phase change during heating. The most plausible explanation is that maghemite and titanomaghemite, which are the low-temperature oxidation product of magnetite/titanomagnetite and commonly found in mafic dykes, were inverted to hematite and (titano)magnetite during heating^32^. We note that some iron sulphides such as pyrrhotite would also breakdown at this temperature interval. However, the presence of pyrrhotite is often characterized by a distinct hump in heating curves, which is not evident in our experiments. Repeated progressive heating experiments^34^ were performed on two representative samples (Fig. 3d and Supplementary Fig. 2f). The results show that two main phase changes occurred at 300–400 °C and 500–600 °C, respectively. The former probably reflects the inversion of maghemite to hematite causing the susceptibility to decline in heating curves, and the latter titanomaghemite inverting to magnetite^32^, responsible for the increase in cooling curves.

IRM acquisition curves (Supplementary Fig. 3) show behaviour consistent with the presence of (titano)magnetite with a rapid increase until saturation at fields of ~100–200 mT. Hysteresis loops show a typical low coercivity behaviour (Supplementary Fig. 4). In a Day plot^35^, the results fall on a MD-SD mixing curve^36^. Moreover, a representative plot of the derivative of the difference of ascending minus descending branch of the positive side of the hysteresis loop reveals two low coercivity peaks (Supplementary Fig. 4).

In summary, our rock magnetic analyses suggest the presence of both MD and SD (low-Ti) titanomagnetite, the latter implying that the BHD dykes are capable of carrying stable magnetic remanence. Additionally, minor amounts of maghemite/titanomaghemite may be present.

### Palaeomagnetism

Two types of thermal demagnetisation behaviour were observed in this study. While ~40% of specimens showed origin-directed stable endpoints, the remaining ~60% revealed only great circle demagnetisation behaviour. For all six dykes, at least one specimen per site yielded stable endpoints. Dyke BHD3 has somewhat random remanence directions, likely caused by the lack of sun compass orientations, which is essential in polar areas so close to the magnetic pole. Circles of confidence for BHD4 and BHD6 site-mean directions are too large (α_95_ > 40°) to place any significance on their directions. We therefore exclude dykes BHD3, BHD4, and BHD6 from further analysis and discussion.

**Figure 4:**
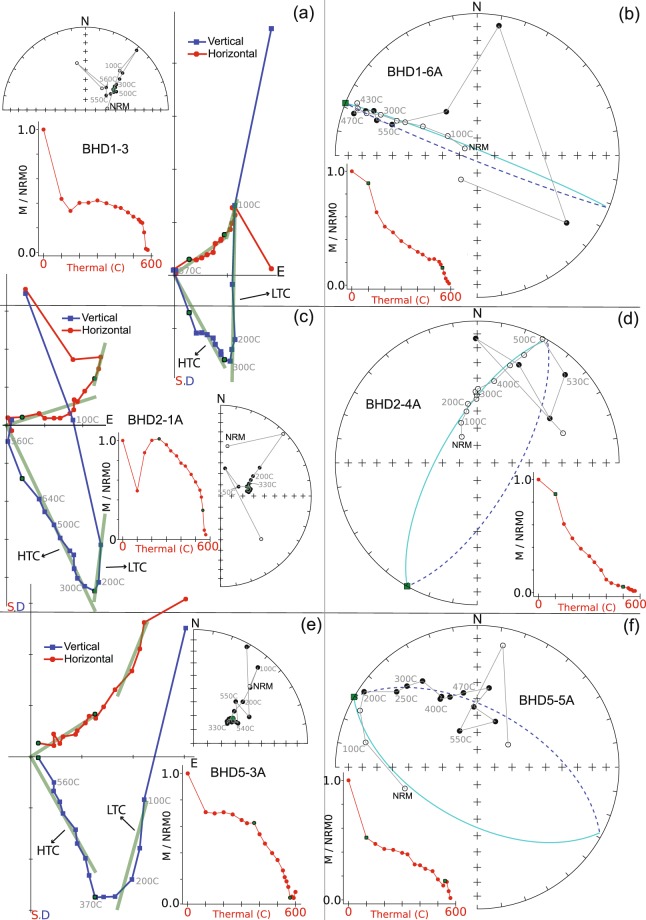
Representative demagnetisation plots. For each site, two specimens are demonstrated: (**a**,**c**,**e**) represent cases with stable endpoints; (**b**,**d**,**f**) represent cases when the stable end points were not reached and the great circle approximations have been made. In equal-area stereonets, open/filled symbols indicate upper/lower hemisphere directions.

**Figure 5:**
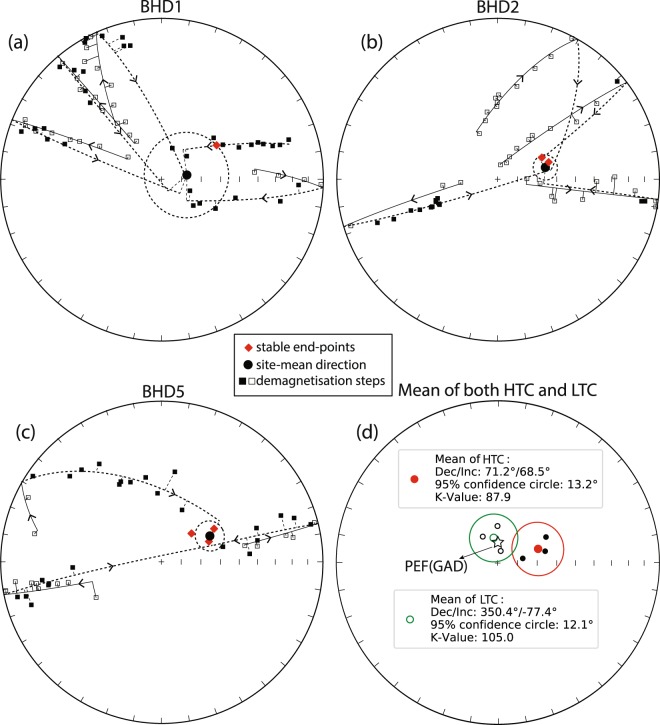
Equal-area stereonets showing the site-mean direction of BHD1, BHD2 and BHD5 as well as the total mean direction of component HTC and LTC. Open/filled symbols indicate upper/lower hemisphere directions. In cases when the stable end points were not reached, all the demagnetisation steps and correspondingly fitted great circles are shown, otherwise only the calculated magnetization vectors are shown.

Thermal demagnetisation of the remaining dykes revealed two single-polarity remanence components based on their unblocking temperatures: a low-temperature component (LTC) and a high-temperature component (HTC, Fig. 4). The LTC is observed in most samples and generally removed by heating to ~250 °C. It is directed steeply upward to the north (*D* = 350°, *I* = −77°, α_95_ = 12°, *k* = 105), which is nearly parallel to the present-day geomagnetic field direction (GAD direction) in the region (Fig. 5d). We interpret the LTC as a viscous remanent magnetisation (VRM) acquired recently. AF demagnetisation was not effective for our sample collection due to a wide scattering of directions after applying alternative fields >50 mT. However, a residual remanence intensity of >10% of the NRM remained even after application of the maximum field (up to 110 mT). This might be explained by a significant population of SD and PSD magnetic carriers, as indicated by the rock magnetic experiments (see previous section).

In cases when magnetisation vectors were defined, the HTC was isolated generally between 370 °C and 530 °C to 570 °C, whereas great circles were calculated using steps between 100 °C and 550 °C. The unblocking temperature range (530–570 °C) suggests low titanium titanomagnetite as the carrier of the HTC. The mean directions defined by intersecting great circles are in good agreement with those by endpoint analyses (Figs 4 and 5), which gives confidence in the method^25^ of mean calculation used for our study. The HTC is thus interpreted to be the characteristic remanent magnetisation (ChRM) which yields a mean direction of *D* = 71°, *I* = 69° (α_95_ = 13°, *k* = 88) (Table 1 and Fig. 5), with a corresponding pole of Plat = −40.5°N, Plong = 150.1°E with A_95_ = 20.3°. Based on our rock magnetic studies and indirect evidence from the AF demagnetisation (see above), we suggest that the HTC is carried by SD or PSD low-titanium titanomagnetite or magnetite, which is palaeomagnetically highly stable (e.g., ref.^31^).

**Table 1:** Palaeomagnetic results for BHD1, BHD2, and BHD5. N/n = number of demagnetised/used samples; Slat, Slong = sample locality latitude and longitude; Decl, Incl = site mean declination, inclination; k = precision parameter of Fisher (1953); α_95_ = radius of cone of 95% confidence; Plat, Plong = latitude, longitude of the palaeopole; Dp, Dm = semi-axes of the cone of confidence about the pole at the 95% probability level.

Site	Trend (°)	N/n	Slat. (°N)	Slong. (°E)	Decl. (°)	Incl. (°)	k	α_95_ (°)	Plat. (°N)	Plong. (°E)	Dp (°)	Dm (°)
BHD1	304	6/6	−66.245278	100.703698	80.5	77.3	15	21.2	−53.8	144.1	37.0	39.6
BHD2	321	6/6	−66.250598	100.622593	76.5	65.4	157	6.0	−37.7	156.7	7.9	9.7
BHD5	300	6/6	−66.215189	100.759104	62.1	62.4	90	7.6	−29.7	148.1	9.2	11.9
Mean		3			71.2	68.5	87.9	13.2	−40.5	150.1	A95 = 20.3°	

Our new palaeomagnetic pole satisfies four out of seven quality criteria of the Q-value of Van der Voo^37^: it is well dated, obtained after an adequate demagnetisation procedure, the studied dykes are post-date the latest stages of the Albany-Fraser Orogeny, so the pole is representative for the Mawson Craton, and finally the pole does not coincide with any younger Antarctic palaeopoles or, after the corresponding Euler rotations, any younger Australian and Gondwanan poles (see syntheses of refs^38–40^ and Supplementary Fig. 5).

In summary, although no baked contact tests are available in this study, several lines of evidence are in favour of a primary origin of the characteristic remanence in the BHD dykes: (i) the presence of SD (titano)magnetite indicates that the BHD dykes are capable of carrying stable magnetic remanence; (ii) the high unblocking temperature between 530 °C and 570 °C makes the HTC unlikely to be affected by a thermal event; (iii) if the Bunger Hills rocks ever experienced remagnetisation, Pan-African orogenesis is the most likely candidate. Nonetheless, the BHD pole does not overlap with poles of Pan-African age or any younger poles (Supplementary Fig. 5), arguing against remagnetisation and for the preservation of primary remanence.

Our pole is calculated by averaging three site-mean directions of three distinct dykes, which may not be enough to average geomagnetic secular variation. More sampling would improve this, but the logistical obstacles are huge for such remote and difficult area as Antarctica. Thus, we assert that the first Precambrian pole from the little-studied Mawson Craton provides an invaluable constraint on Precambrian palaeogeography and tectonics, which we demonstrate in the next section.

## Discussion

East Antarctica represents the Precambrian portion of Antarctica, and most workers agree that it is divisible into several tectonic domains that have geological affinities with Africa (Kalahari), India, Australia, and some unknown sources^7,8,16,18,41,42^. Antarctic rocks with Australian affinities are often considered to have been connected with Australia until the breakup of Pangaea, which commenced at ~85 Ma (e.g., ref.^43^). Various terms have been used to describe the once contiguous Australia-Antarctica continental block. For the purposes of this paper, we use the term “the Mawson Craton” first used in refs^38,39^. The extent of the Mawson Craton is unclear due to extensive ice cover (and unlike West Antarctica that is melting rapidly, the East Antarctic ice sheet remains stable or is possibly even gaining mass^44^). Here we follow the continental outline of refs^7,11,18^, and consider that the Mawson Craton (comprised by Terre Adélie terrane, Miller Range, and other tectonic units surrounding them) has been connected with the Gawler Craton of Australia in the so-called Mawsonland configuration (Fig. 1) since Archaean. Note that we do not include Wilkes Land (including Bunger Hills and Windmill Islands), which were traditionally considered parts of the Mawson Craton, because we only show the outline of the Mawson Craton before the Albany-Fraser Orogeny (Fig. 1).

Although it is generally agreed that Precambrian Australia (west of the Tasman line; Fig. 1) is composed of three Archaean to Palaeoproterozoic cratons (the West, North, and South Australian cratons – WAC, NAC and SAC correspondingly), when and how the present-day configuration took form is still a matter of debate. The amalgamation between the NAC and WAC were originally thought to have taken place during the ca. 1800–1765 Ma Yapungku Orogeny^45–47^. However, the relatively high-pressure metamorphism presumably reflecting the collision between of the WAC and NAC was recently suggested to have possibly occurred as late as ca. 1300 Ma^48,49^, in favour of a late assembly between WAC and NAC. The relationship between the NAC and SAC is even more intensely debated. Based mainly on the similarity between the Mount Isa Terrane of the NAC and the Curnamona Province of the SAC, most recent models^46,47,50,51^ propose that the SAC was connected with the NAC from at least ca. 1800 Ma until they broke apart ca. 1500 Ma. The SAC then reunited with the NAC during the ca 1330–1140 Ma^17^ Albany-Fraser Orogeny in a different configuration.

**Figure 6:**
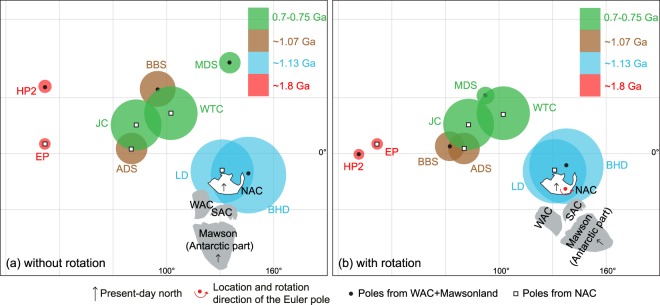
Four groups of coeval poles (Table 2) from the WAC + Mawson and NAC plotted in Mercator projection. Mawson (Antarctic Part) rotated to SAC in its Gondwana configuration using a Euler pole^7^ at 1.3°N, 37.7°E, rotation = 30.3°. (**a**) Australia in its present-day configuration; (**b**) WAC + SAC + Mawson rotated to NAC about a Euler pole^54^ at 20°S, 135°E, rotation = 40°.

In spite of all the disputes, nearly all proposed models (e.g., refs^46,50–53^) share some common ground in that the previously combined WAC + NAC amalgamated with the SAC (together with the Mawson Craton) forming Precambrian Australia by the end of the Albany-Fraser Orogeny ca. 1140 Ma^17^. This amalgamation allows Mawson + Australia to be viewed as a single continental block in post-1.2 Ga reconstructions (e.g., refs^12,17,41^). However, such an early formation of the present-day cratonic Australia cannot explain apparent mismatches between some coeval palaeomagnetic poles of Australia, exemplified by the ~35° discrepancy between the 1070 Ma Bangemall Basin sills (BBS) pole of the WAC and the 1070 Ma Alcurra dykes and sills (ADS) pole of the NAC (Fig. 6; ref.^54^).

To address such mismatches between coeval poles within Australia, one solution is to have major Australian cratons not assembled until after ~1070 Ma^55^. In Fig. 6a, selected palaeomagnetic poles (Table 2) including the Bunger Hills dykes pole (BHD) were used to test this hypothesis of a late Australian amalgamation. The BHD pole and that of the ca. 1140 Ma Lakeview dolerite of the NAC overlap, implying that the collision of WAC + SAC + Mawson with NAC finished or at least was close to suturing by ca. 1133 Ma, which is inconsistent with the post-1070 Ma assembly of Australia^55^. Additionally, the coherent ca. 800–600 Ma Centralian Superbasin stratigraphy makes it geologically unfeasible to close putative wide late Neoproterozoic ocean basins to form Australia^54^.

**Table 2:** Palaeomagnetic poles used in this study.

Pole	Abbr.	Plat. (°N)	Plong. (°E)	A_95_ (°)	Age (Ma)	Reference
**North Australian Craton**						
Elgee-Pentecost Formations	EP	5.4	31.8	3.4	1803–1793	^45, 77, 78^
Lakeview dolerite	LD	−9.5	131.1	17.4	1147–1135	^79^
Alcurra dykes and sills	ADS	2.8	80.4	8.8	1087–1066	^55^
Johnny’s Creek Member (Bitter Springs Formation)	JC	15.8	83.0	13.5	780–760	^56^
Walsh Tillite Cap Dolomite	WTD	21.5	102.4	13.7	750–700	^80^
**West Australian + Mawson cratons**						
Hamersley Overprint 2	HP2	8.0	338.0	5.0	~1800	^81^
Bunger Hills dykes*	BHD	−11.9	145.5	20.3	1134–1131	This study
Bangemall Basin sills	BBS	33.8	95.0	8.3	1076–1066	^61^
Mundine Well dykes	MDS	45.3	135.4	4.1	758–752	^82^

^*^Rotated to West Australia Craton using the Euler pole^7^ at 1.3°N, 37.7°E, rotation = 30.3° .

An alternative solution is that the WAC + SAC rotated ~40° with respect to the NAC ca. 650–550 Ma^54^, which was argued on the basis that such an intraplate rotation brings three pairs of coeval, previously discrepant poles into agreement. A new pole from the ca. 770 Ma Johnny’s Creek Member (Bitter Springs Formation) lends further support for this intraplate rotation^56^. The BHD and LD poles make up another group of coeval poles from the NAC and WAC + SAC + Mawson, respectively, with which the intraplate rotation may be further tested. With the rotation applied, the area of overlap of the 95% confidence circles of the BHD and LD poles increases (Fig. 6), which provides a positive test for the relative rotation model between WAC + SAC(+Mawson) and NAC. The vast intracratonic rotation hypothesis not only reconciles discrepant coeval palaeopoles, but also provides a mechanism for the enigmatic Paterson and Petermann orogenies that accounts for significant mineralisation such as the massive Telfer Au deposit^57,58^.

Given the coincidence of the coeval BHD and LD poles when restored to the earlier Proterozoic configuration of Australia (Fig. 6b), we calculate a mean ca. 1134 Ma pole for Australia + Mawson. This mean pole calculation thus overcomes the shortcoming of the BHD pole potentially undersampling geomagnetic secular variation. Calculation is conducted by combining the individual virtual geomagnetic poles of both the LD and BHD studies using Fisher statistics after rotating the BHD data into the North Australia reference frame according to the Euler parameters in ref.^54^. The resultant ca. 1134 Ma mean pole for Australia + Mawson (in North Australian coordinates) is 9°S, 134°E and A95 = 14°.

**Figure 7:**
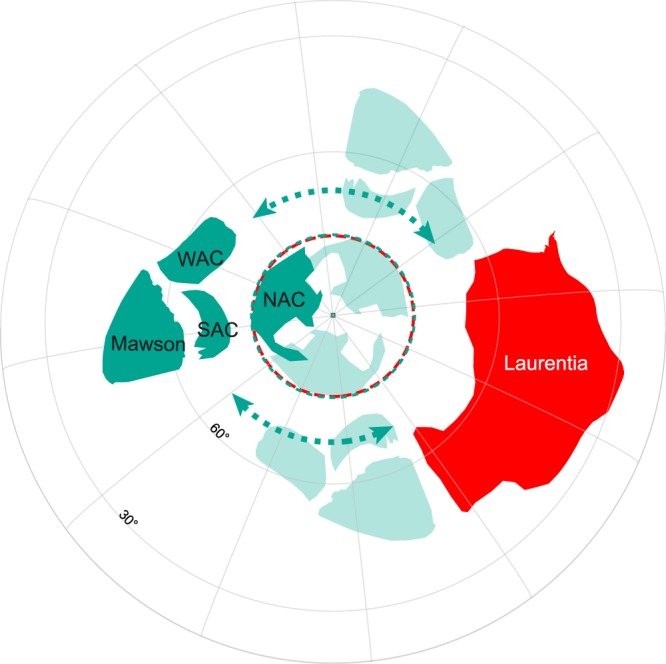
Possible positions of Australia + Mawson (green) relative to Laurentia (red) ca. 1134 Ma. Relative palaeolongitude is unconstrained by such a single-pole comparison, indicated by arrow ranges and three possible positions of Australia depicted relative to Laurentia. The preferred Australian option (dark shading) makes a SWEAT-like fit easily achievable both before (supercontinent Nuna) and after (supercontinent Rodinia) this time of separation between Laurentia and Australia + Mawson. Other options depicted (light shading) get Australia-Mawson closer to Laurentia, but in configurations significantly different than SWEAT. Absolute palaeolongitude of Laurentia is arbitrary and unlabelled.

The combined, and therefore time-averaged, ca. 1134 Ma pole for Australia + Mawson can be used for robust palaeogeographic reconstruction and we do so here to test the SWEAT (Southwest US-East Antarctic) fit, which is probably the best-known and most-debated relationship in Precambrian supercontinents. Figure 7 demonstrates that the SWEAT fit requires some space between Laurentia and Australia + Mawson even when adopted the so-called “closest approach”^59,60^. Our comparison (Fig. 7), as with previous studies^61–63^, suggest that the SWEAT fit was not viable between ca. 1210 Ma and ca. 1070 Ma. If SWEAT-like fits did indeed exist in both Nuna^2,4,64–66^ and Rodinia^67–70^, then Australia + Mawson must have rifted away from Laurentia during Nuna breakup^2,4,71^, but likely remained close for later assembly in Rodinia in a broadly similar configuration^72^.

**Figure 8:**
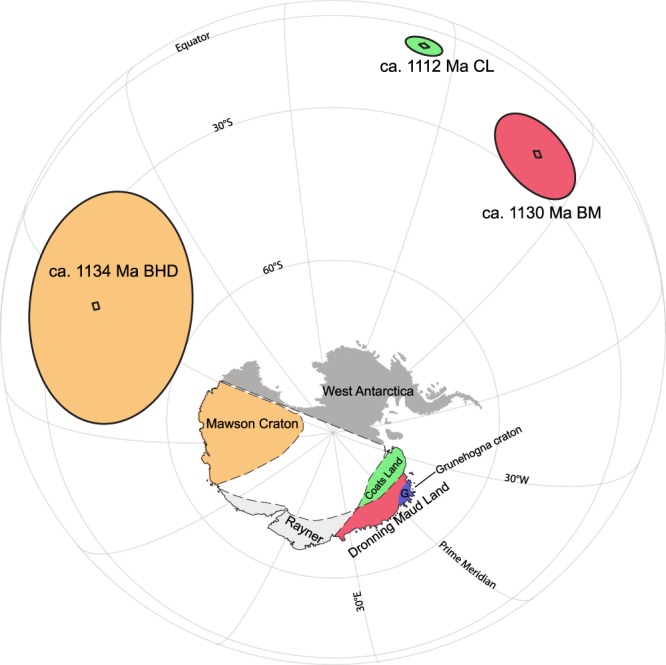
The BHD pole from this study plotted with the only other two extant palaeomagnetic poles from East Antarctica. Palaeomagnetic poles are colour-coded to the continental blocks from which they derive. Abbreviations: BHD, Bunger Hills dykes; BM, Borgmassivet intrusions; CL, Coats Land.

Lastly, the new BHD pole presented here also carries implications for the amalgamation of Antarctica. Grenville-age orogenic belts (ca. 1.1 Ga) surrounding East Antarctica were thought to comprise one continuous belt, implying that the East Antarctica had already formed, (e.g., refs^57,59^) until a geochronology study^8^ differentiated three distinct provinces on the basis of U-Pb zircon data. The disagreement of the BHD pole and the only other two existing and roughly coeval poles(Fig. 8) from East Antarctica^5,6^ suggests that the Dronning Maud Land and Coats Land regions were not rigidly connected to the Mawson Craton ca. 1134 Ma, confirming the hypothesis of ref.^8^. Coats Land was originally considered to be the extension of the Grenville orogen into East Antarctica in Rodinia and thus in support of the SWEAT connection^69^. A paleomagnetic study^6^ suggested that Coats Land might actually have belonged to the Kalahari Craton and far from the East Antarctica at ca. 1.1 Ga despite the 30° difference between the CL pole and the roughly coeval poles of Kalahari. Subsequent studies^10,73^, however, showed that Coats Land was neither part of Kalahari nor East Antarctica ca. 1.1 Ga. Instead, Coats Land as part of Laurentia collided with Dronning Maud Land (specifically the Grunehogna Craton), which was widely accepted as piece of the Kalahari (see refs^9,74^ for example) before joining East Antarctica, along the ca. 1090–1060 Ma Maud Belt during the formation of Rodinia. Kalahari, with Coats Land attached to it, then collided with East Antarctica along the East African-Antarctic Orogen ca. 650–500 Ma within an assembling Gondwana. The succeeding Mesozoic breakup of Gondwana stripped Coats Land and Dronning Maud Land away from Kalahari and abandoned them in East Antarctica.

## Conclusion

A pilot palaeomagnetic study in the Bunger Hills has attained the first Precambrian palaeopole for the Mawson Craton. Palaeomagnetism of the Bunger Hills dykes supports the vast late Neoproterozoic relative rotation between the NAC and the WAC + Mawson. Mean pole calculation (BHD-LD) allows comparison between Australia-Mawson and the coeval Abitibi dykes pole of Laurentia and demonstrates, as with previous studies, that the SWEAT fit is not viable between ca. 1210 Ma and ca. 1070 Ma. Comparison between the BHD, BM, and CL poles confirms that the Grenville-age ca. 1.1 Ga orogenic belts surrounding the East Antarctic coastline do not constitute a continuous orogenic belt.

## Electronic supplementary material

Supplementary information
