# Supplementary material for: First Precambrian palaeomagnetic data from the Mawson Craton (East Antarctica) and tectonic implications
Source: Sci Rep. 2018 Nov 6;8:16403. doi: 10.1038/s41598-018-34748-2 (PMC6219563; doi:10.1038/s41598-018-34748-2)
Supplement: Supplementary file 1 — Supplementary information [file 41598_2018_34748_MOESM1_ESM.docx]

Supplementary information for:

**First Precambrian palaeomagnetic data from the Mawson Craton (East Antarctica) and tectonic implications**

**Yebo Liu1*, Zheng-Xiang Li1, Sergei A. Pisarevsky1, Uwe Kirscher1, Ross N. Mitchell^1^, J. Camilla Stark^1^, Chris Clark^2^, Martin Hand^3^**

1. *Earth Dynamics Research Group, ARC Centre of Excellence for Core to Crust Fluid Systems (CCFS) and The Institute for Geoscience Research (TIGeR), School of Earth and Planetary Sciences, Curtin University,* *GPO Box U1987, WA 6845, Australia*
2. *The Institute for Geoscience Research (TIGeR), School of Earth and Planetary Sciences, Curtin University, GPO Box U1987, WA 6845, Australia*
3. *Department of Earth Science, School of Physical Sciences, University of Adelaide, Adelaide, South Australia 5005, Australia*


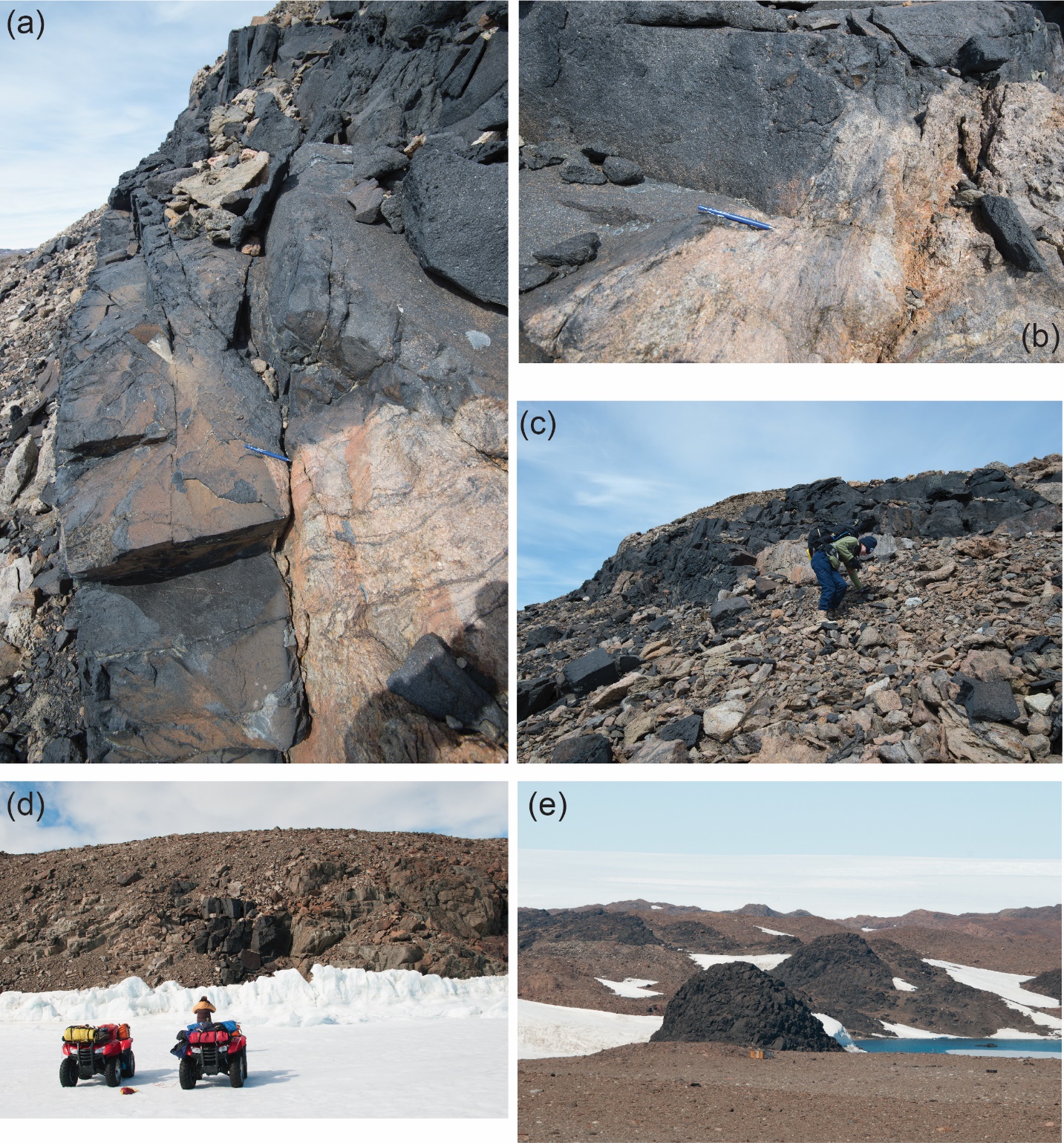


**Supplementary Figure 1. Field photos for the Bunger Hills mafic dykes. (a), (b) and (C)** BHD1, **(d)** BHD3, **(e)** BHD4


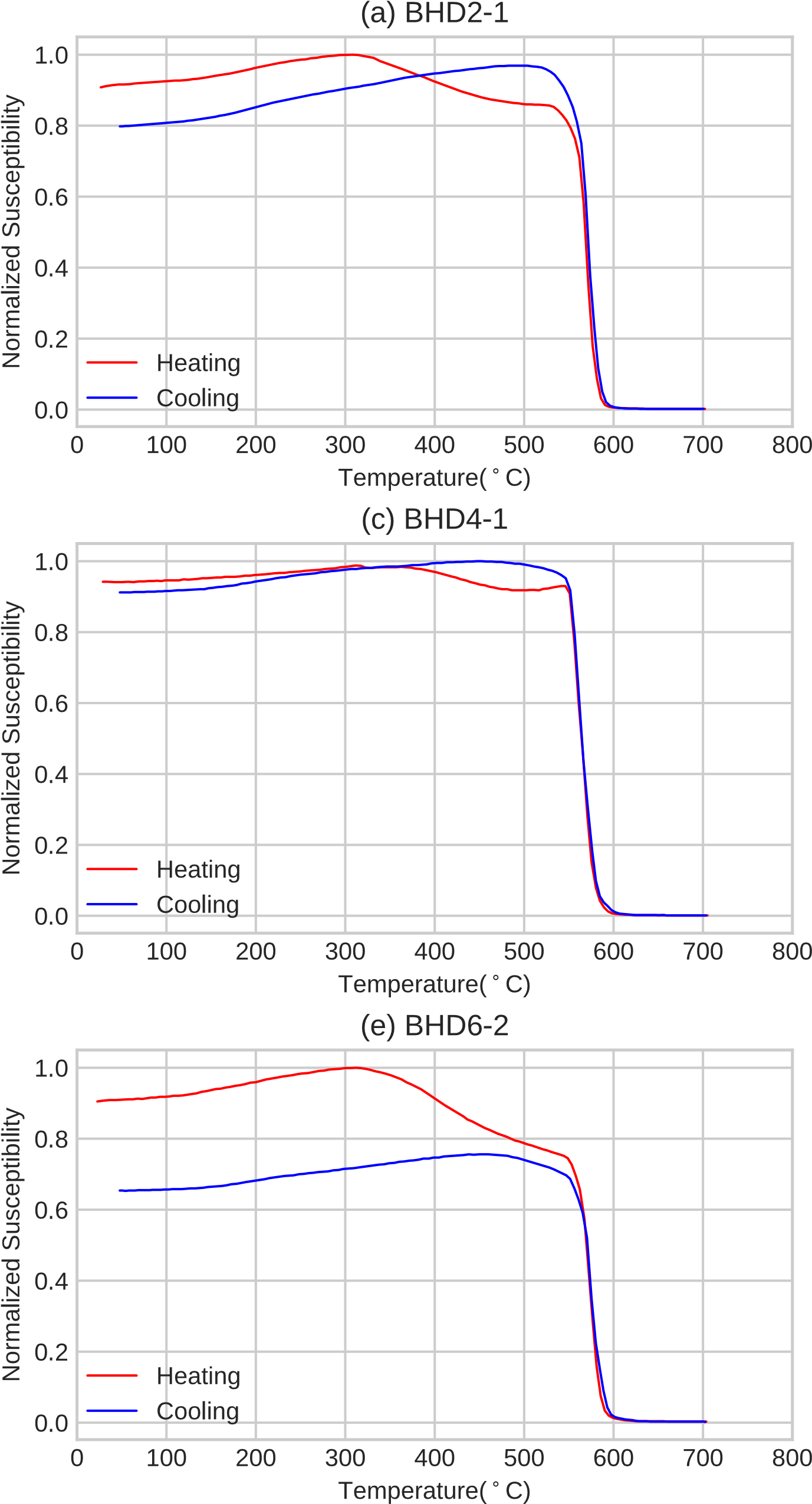

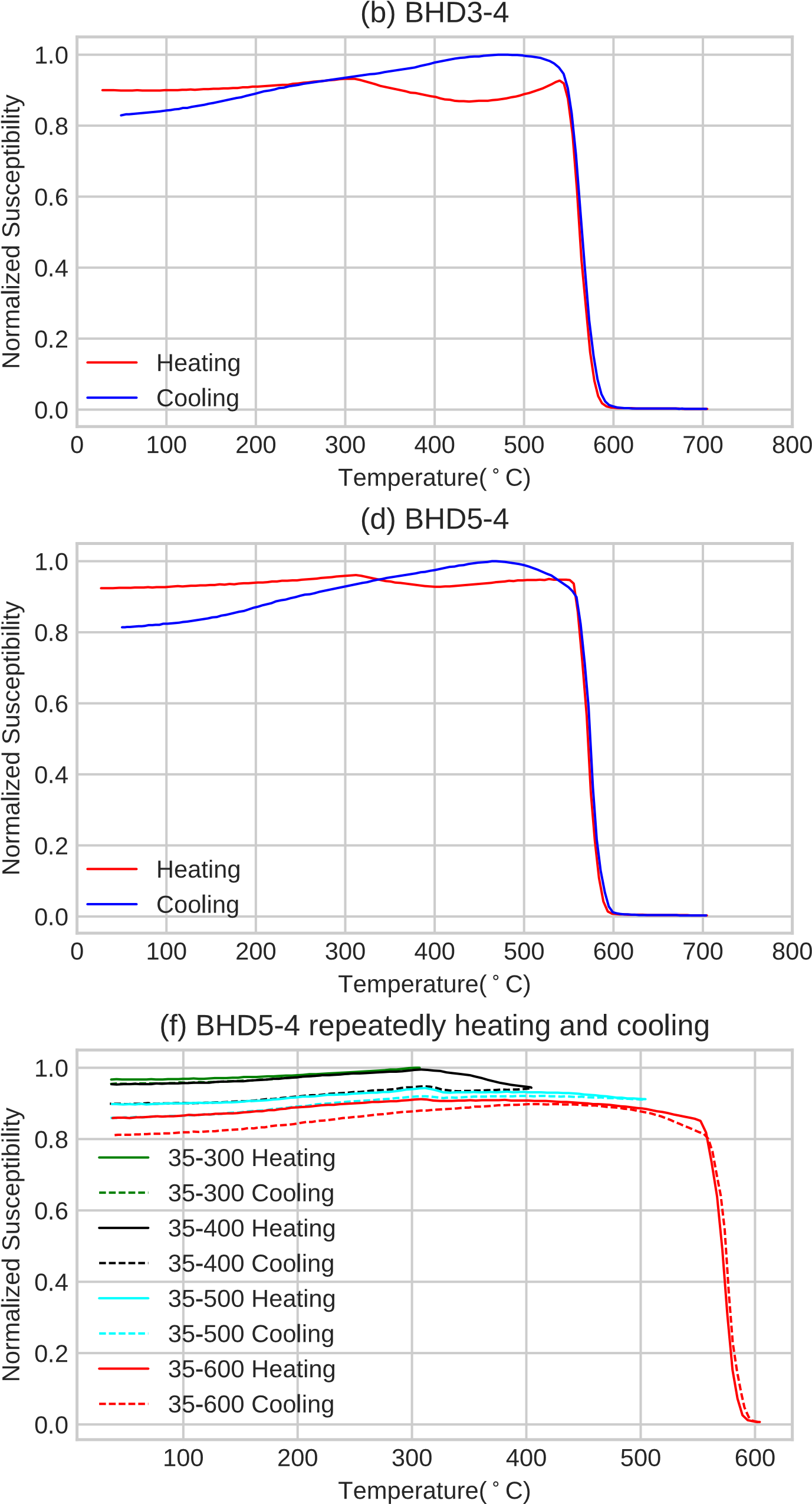


**Supplementary Figure 2. Susceptibility versus temperature data for representative Bunger Hills dyke samples.**


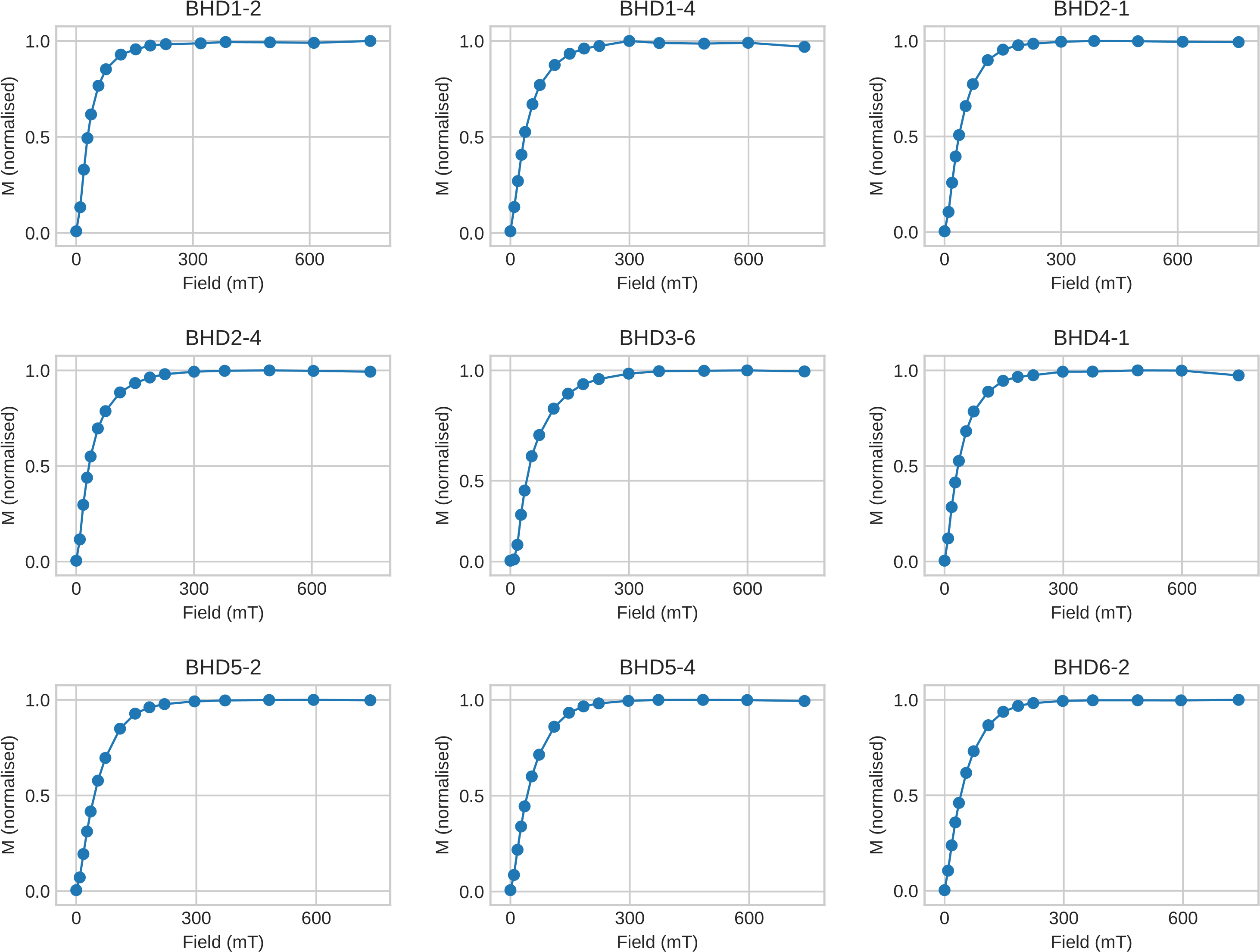


**Supplementary Figure 3. Normalized IRM acquisition curves for Bunger Hills dyke samples.**


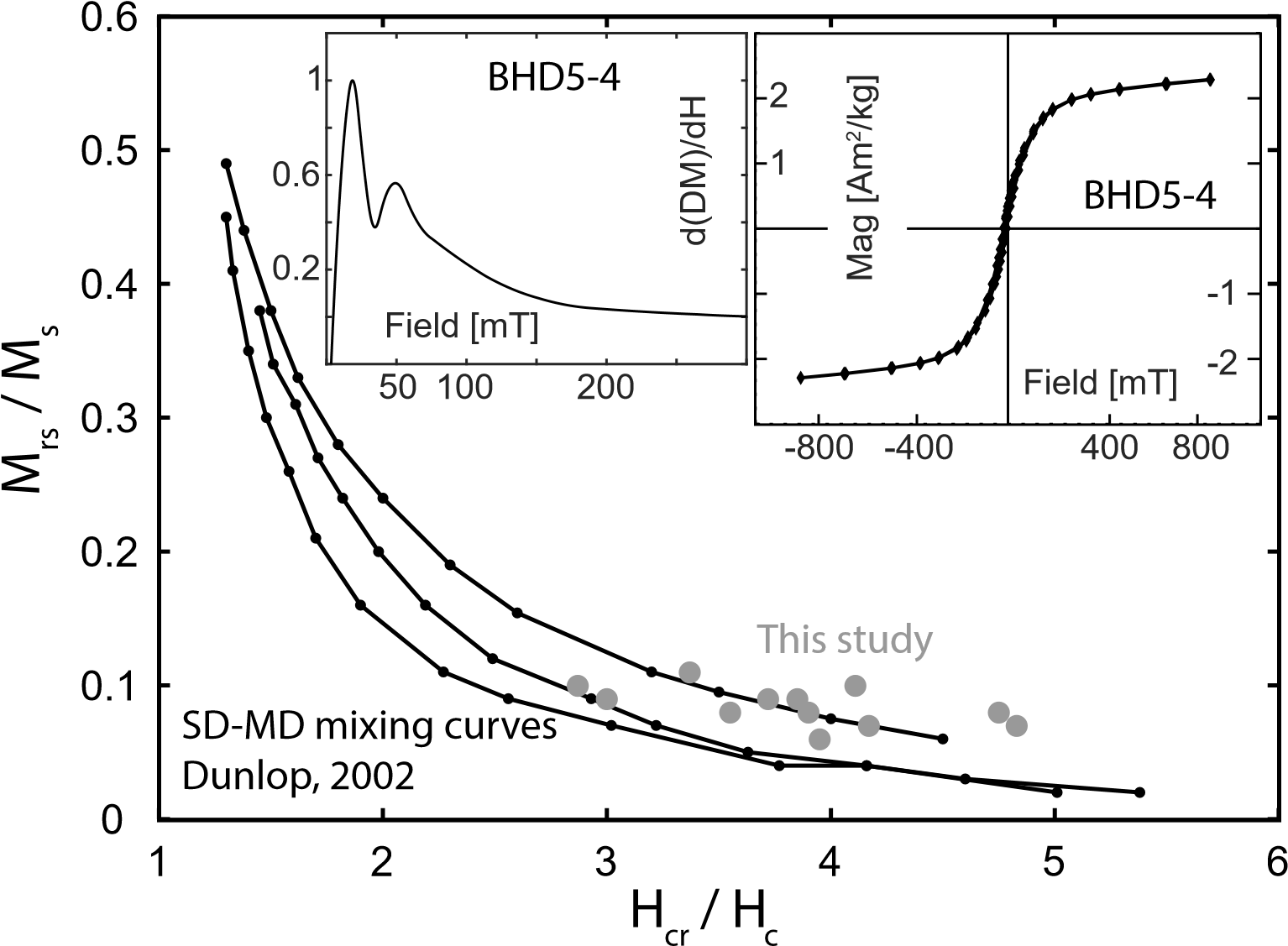


**Supplementary Figure 4. Day plot**^1^ **and hysteresis loops of representative Bunger Hills dyke samples. Also shown are theoretical SD-MD mixing curves**^2^**. Upper left inset shows the derivative of the difference of ascending minus descending branch of the positive side of the hysteresis loop**^3^**.**


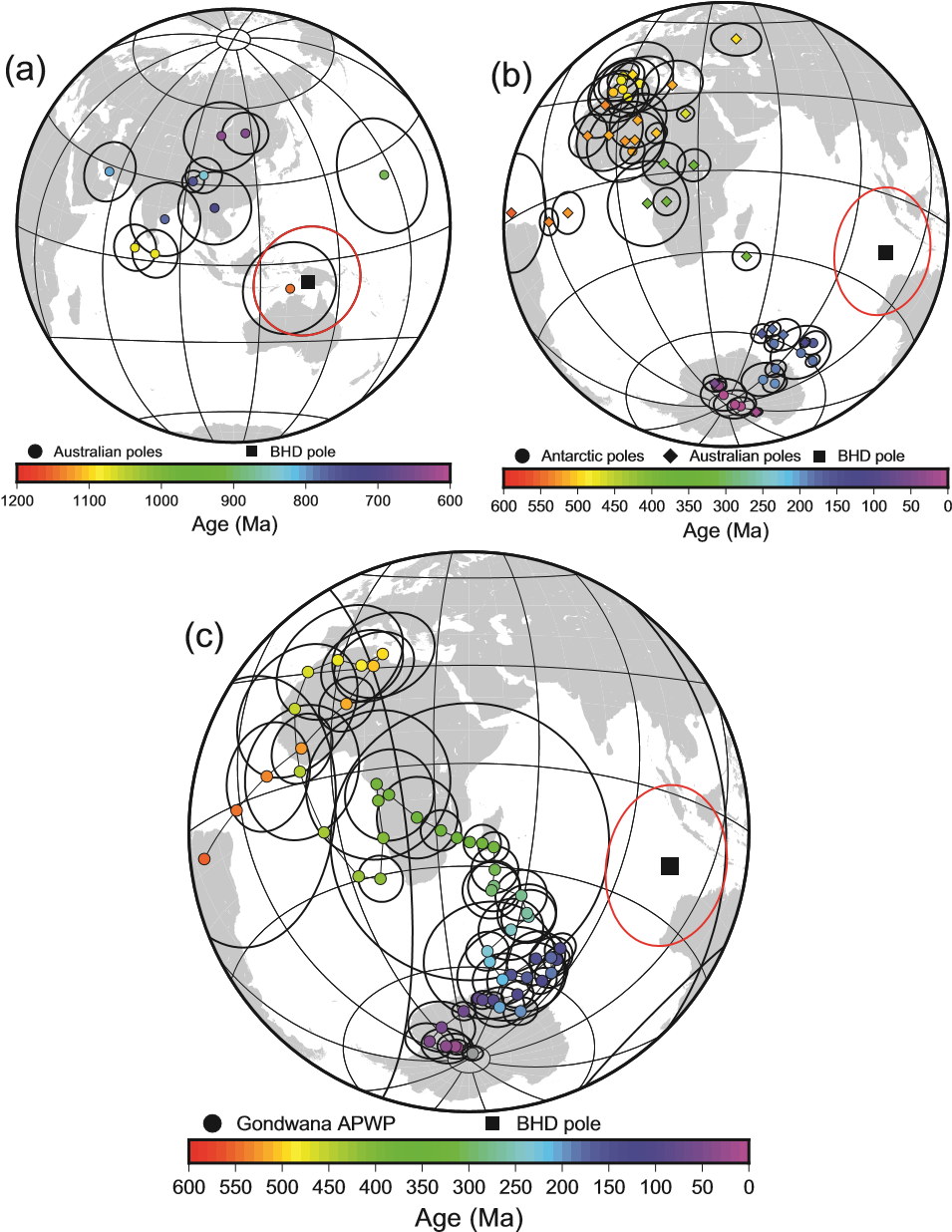


**Supplementary Figure 5. BHD pole compared with younger poles. (a) BHD pole and younger Precambrian Australian poles in North Australia coordinates**. BHD pole is first rotated to Western Australia coordinates using a Euler pole^4^ at 1.3°N, 37.7°E, rotation = 30.3°, then, together with the WAC+SAC poles^5^, rotated to North Australia coordinates using a Euler pole^6^ at 20°S, 135°E, rotation = 40°; **(b) BHD pole and Phanerozoic poles from Australia and Antarctica in South Africa coordinates**. BHD pole is rotated to South Africa coordinates using a Euler pole at 10.4°N, 148.7°E, rotation=-58.4°. Australia and Antarctica poles are from the compilation of ref. ^7^; **(c) BHD pole and Gondwana APWP**^7^ **in South Africa coordinates.**

**References**

1. Day, R., Fuller, M. & Schmidt, V. A. Hysteresis properties of titanomagnetites: Grain-size and compositional dependence. *Phys. Earth Planet. Inter.* **13,** 260–267 (1977).

2. Dunlop, D. J. Theory and application of the Day plot ( M rs / M s versus H cr / H c ) 1. Theoretical curves and tests using titanomagnetite data. *J. Geophys. Res.* **107,** 2056 (2002).

3. Tauxe, L., Mullender, T. A. T. & Pick, T. Potbellies, wasp-waists, and superparamagnetism in magnetic hysteresis. *J. Geophys. Res. Solid Earth* **101,** 571–583 (1996).

4. Collins, A. S. & Pisarevsky, S. A. Amalgamating eastern Gondwana: The evolution of the Circum-Indian Orogens. *Earth-Science Rev.* **71,** 229–270 (2005).

5. Schmidt, P. W. A review of Precambrian palaeomagnetism of Australia: Palaeogeography, supercontinents, glaciations and true polar wander. *Gondwana Res.* **25,** 1164–1185 (2014).

6. Li, Z. X. & Evans, D. A. D. Late Neoproterozoic 40° intraplate rotation within Australia allows for a tighter-fitting and longer-lasting Rodinia. *Geology* **39,** 39–42 (2011).

7. Torsvik, T. H. *et al.* Phanerozoic Polar Wander, Palaeogeography and Dynamics. *Earth-Science Rev.* **114,** 325–368 (2012).
